# Department of Surgery

**Published:** 1900-08

**Authors:** W. E. A. Wyman, J. A. Sloan, Robert Jay

**Affiliations:** Burlington, Wis.; San Francisco, Cal.; West Liberty, Ia.


### DEPARTMENT OF SURGERY.

BY W. E. A. WYMAN, M.D.V., V.S.,
BURLINGTON, WIS.

J. A. SLOAN, B.SC., M.D.V., SAN FRANCISCO, CAL.

DR. ROBERT JAY, M.D.V., WEST LIBERTY, IA.

### SURGERY OF THE PENIS.

PHIMOSIS. More or less stenosis of the preputial opening, interfering with the protrusion of the penis, is termed phimosis.

This condition may be congenital or it may be acquired, the latter the more frequent one. In geldings where, as the result of castration, atrophy of the penis is seen, phimosis—due to the irritating influence of decomposing urine in the preputial cavity, causing at first an acrobustitis—is likely to occur. Other agents causing phimosis are: neoplasms; undue accumulation of preputial smegma; the empirical introduction of pepper, etc.; traumatic insults, as kicks.

At first, slight œdema of the sheath, painful to the touch, is noticed. On introducing the hand into the preputial cavity, accumulated and hardened, badly-smelling sebaceous matter is met, the head of the penis feeling hot and painful. In stallions the pain is very evident during an erection. At times there is complete cessation of micturition, accompanied by colicky symptoms of course. In those cases there is usually a blockage, due to accumulated secretions in the urethral sinus (a cavity beneath the urethral tube). These accumulated secretions are termed vulgarly "beans." As a rule, this condition terminates favorably; nevertheless, a urethritis or abscess-formations in the sheath are possibilities.

The treatment of congenital phimosis is a surgical one. The preputial opening is simply incised until proper dimensions exist, it being unquestionably well to cauterize the edges of the wounds to retard healing and prevent too vigorous scar-tissue formation, or, in other words, another stricture, which again would have to be split. Personally, I prefer the cautery for this purpose. Thorough cleansing of the field of operation is essential before any surgical steps are taken, and hardly requires mention. As the preliminary steps: Soak with warm soapsuds; remove all accumulations; irrigate with a 1 per cent. solution of formalin or 2 per cent. germol, chloronaptholeum, creolin, etc., After the operation irrigate daily with some antiseptic. I give a 2 per cent. alum solution the preference.

Warts, new-growths of a malignant type, papillomata, etc., constituting a cause of phimosis, are to be removed in the ordinary way. Multiple neoplasmata, some of which cannot be reached from the preputial opening, I have removed by splitting the sheath in the median line, thus obtaining an excellent field for the operation. Such a wound is treated in no way different from ordinary incised wounds.

Paraphimosis. Whenever the penis cannot be retracted, or only partly retracted into the sheath, due to a narrowing of the preputial opening, the state of paraphimosis exists. Traumatism is the most frequent cause; undue swelling following castration; coitus; irritants, such as pepper, introduced into the sheath; finally, new-growths on the glans penis, preventing its retraction.

The swollen sheath with the free portion of the penis hanging from it attract attention. Depending on the amount of time passed, the inflammatory infiltration into the parts eventually encircles and grasps the penis, as a result of which circulatory disturbances set in, the head of the penis swells, is very painful, and appears reddened. Even cases which at first sight appear as serious ones, unless paralysis of the penis aggravates the condition, respond very kindly to treatment.

A great many agents are recommended: Massage of the parts; bandaging of the penis, and keeping the bandage moist with astringents, such as alum, tannic acid, etc. Of the various therapeutic resources, the following has given me excellent results, especially in bad cases which appeared quite hopeless, and great improvement was visible within twenty-four hours; therefore, I suggest its use primarily in cases where inflammatory infiltration is well advanced. An ointment of one part cantharides vesic. and eleven parts of adepis is rubbed into the diseased parts. Four to six hours later, when vesication is established, slight scarification all around the circumference of the stenosed preputial opening is executed. As previously stated, the good results of this mode of treatment manifest themselves within twenty-four hours. In all cases of some moment a suspensory is, in my opinion, a valuable therapeutic associate. Should paraphimosis be due to new-growths on the penis, the causa belli, the neoplasm, must be removed. As a last resource, when all other means have failed, the amputation of the penis may be practised.

Wounds of the Prepuce. More or less hemorrhage due to injury of branches of the subcutaneous abdominal artery may be present. Barb-wire cuts, quite frequently seen in the country, may even penetrate through all the layers of the sheath, even wounding the penis. Kicks and pointed objects may also give rise to wounds. Hemorrhage within the sheath may cause urinary retention, the coagulated blood preventing the flow of urine. Unless phimosis, due to a stricture, depending on scar-tissue formation, is likely to follow, the prognosis is good, as most lesions respond readily to treatment.

In regard to the treatment, it is essential to stop the hemorrhage, either by ligating the vessels, should some of the larger ones have been cut, or by bringing the edges of the wound together by closely inserted stitches. Even in lacerated wounds it is well to sew, as the wound gaps less subsequently. Foreign bodies, as splinters, glass, etc., are necessarily to be removed before the active treatment is begun. Antiseptic washes conclude the treatment. The stitches are to be removed between the fourth and sixth day, when slight sloughing first shows itself.

A very interesting case of phimosis, in a yearling colt, due to a penetrating barb-wire cut across the whole of the sheath, I saw through the courtesy of my neighbor, Dr. F. Moyle, of Waterford, Wis. Dr. Moyle says the prepuce was divided at a right angle to the linea alba. The wound was treated according to the recognized rules of surgery and the possibility of phimosis prognosed. Eventually the stricture became so well developed that even the small finger could not be passed. To do away with acrobustitis and balanitis, the opening was enlarged with a probe-pointed bistoury, the edges of the wound cauterized, and antiseptic irrigations ordered. The penis at the time the operation was performed was about the size of a little finger, having atrophied to that extent.

Wounds of the Penis. The pendulous portion of the penis is more exposed to injuries than the fixed portion, and owing to the rich blood-supply hemorrhage is an essential factor. Depending on the intensity of the lesion, the prognosis would change. Thus in superficial wounds it is good; but when the urethra is injured, possibly cut into, a guarded prognosis is imperative. The causes are projecting nails, barb-wire, kicks from ticklish mares, violent coitus, etc., as in mares which are not horsing.

The symptoms are well marked. The penis may hang out of the sheath, and in serious cases paralysis is present. Enormous swellings in the scrotal region, even as far as the thighs, due to infiltration of blood, may be noticed; such complications as paraphimosis, balantitis, paralysis of the penis, even suppression of the urine, may occur.

In superficial wounds antiseptic irrigations are sufficient. When the urethra is severed, closely inserted catgut stitches are indicated as well as permanent catherization until healing has taken place to prevent urethral stenosis. Two cases treated by the writer in this manner made an apparent recovery, to develop, within four months after removal of the catheter, a stricture which eventually led to amputation of the penis. The stricture was treated at first with dilators, but the animals became so vicious that a long story was shortened by amputating the penis just above the stricture. I have never found it necessary to ligate the arteries as the hemorrhage subsided as soon as the sutures were put in. Should there be marked infiltration of blood, due to stasis in the penis, a pressure-bandage is of use.

Of course, in those cases where the penis is practically cut into, amputation of the severed part becomes necessary. In making the prognosis it is well to remember that in all wounds of the penis accompanied by loss of tissue the shape of the penis is likely to become changed, due to scar-tissue formation; thus the penis may become crooked, slightly twisted or bent.

In conclusion, bruises of the tissues of the penis are of interest. Stallions, when kicked on the penis during its state of erection, often show symptoms which are alarming. As a result of the extravasation of blood into the spongy portion of the corpus cavernosum an enormous swelling of the penis occurs. The tumor is soft, very painful and fluctuates. Phimosis or paraphimosis may be present. Ordinary contusions of the penis terminate by resolution.

As regards the hæmatoma of the penis, alluded to above, the main treatment is to leave it alone, and with due respect to the knife the surgeon loves so well, do not cut. The reasons are: Early puncture of a hæmatoma is very likely to be followed by serious hemorrhage and also infection. In three or four days—in between using cooling irrigations, followed immediately by light exercise and a suspensory—the remains of the tumor may be freed by an incision, and the cavity flushed with some antiseptic solutions regularly after that. When puncturing the tumor on the inferior face of the penis care should be taken to avoid injury to the urethra.

